# Identification of a major QTL and candidate genes analysis for branch angle in rapeseed (*Brassica napus* L.) using QTL-seq and RNA-seq

**DOI:** 10.3389/fpls.2024.1340892

**Published:** 2024-02-21

**Authors:** Shaolin Lei, Li Chen, Fenghao Liang, Yuling Zhang, Chao Zhang, Huagui Xiao, Rong Tang, Bin Yang, Lulu Wang, Huanhuan Jiang

**Affiliations:** ^1^ Guizhou Oil Crops Research Institute, Guizhou Academy of Agricultural Sciences, Guiyang, Guizhou, China; ^2^ Guizhou Rapeseed Research Institute, Guizhou Academy of Agricultural Sciences, Guiyang, Guizhou, China

**Keywords:** rapeseed, branch angle, BSA-seq, QTL mapping, RNA-Seq

## Abstract

**Introduction:**

Branching angle is an essential trait in determining the planting density of rapeseed (*Brassica napus* L.) and hence the yield per unit area. However, the mechanism of branching angle formation in rapeseed is not well understood.

**Methods:**

In this study, two rapeseed germplasm with extreme branching angles were used to construct an F_2_ segregating population; then bulked segregant analysis sequencing (BSA-seq) and quantitative trait loci (QTL) mapping were utilized to localize branching anglerelated loci and combined with transcriptome sequencing (RNA-seq) and quantitative real-time PCR (qPCR) for candidate gene mining

**Results and discussion:**

A branching angle-associated quantitative trait loci (QTL) was mapped on chromosome C3 (C3: 1.54-2.65 Mb) by combining BSA-seq as well as traditional QTL mapping. A total of 54 genes had SNP/Indel variants within the QTL interval were identified. Further, RNA-seq of the two parents revealed that 12 of the 54 genes were differentially expressed between the two parents. Finally, we further validated the differentially expressed genes using qPCR and found that six of them presented consistent differential expression in all small branching angle samples and large branching angles, and thus were considered as candidate genes related to branching angles in rapeseed. Our results introduce new candidate genes for the regulation of branching angle formation in rapeseed, and provide an important reference for the subsequent exploration of its formation mechanism.

## Background

*Brassica napus* is an important oil crop worldwide. The yield is the ultimate goal of rapeseed genetic improvement. With the increasing world population and decreasing availability of cultivated land, high planting density has become crucial to ensure food security. To adapt to high intensity cultivation, many agronomic traits of crops have been changed in the process of breeding and domestication, but the change in plant type is the most prominent (Duvick, 2005). Plant type refers to the spatial distribution of the plant organs (Teichmann and Muhr, 2015). Ideal Plant Architecture (IPA) can improve the photosynthetic efficiency of crop and increase dry matter accumulation (Zhang et al., 2017). Studies have shown that a moderately compact plant type is conducive to ventilation and light transmission in the middle and lower parts of *B. napus*, and can reduce pests and diseases while enhancing lodging resistance (Hu et al., 2017). The branch angle is a primary index for determining the looseness and compactness of rapeseed; therefore, the improvement of the branch angle has become an important breeding goal for *B. napus*.

Many studies have shown that the branch (tiller) angle is a quantitative trait, controlled by major genes and modified by minor genes (Yu et al., 2007; Liu et al., 2016; Clark et al., 2022; Liu et al., 2022). The important factors affecting branch (tiller) angle are genetics, hormone regulation, and environmental factors (such as planting density) (Wang et al., 2022a). *PROG1*, a zinc finger transcription factor cloned from rice, influences both tiller number and tiller angle and plays a pivotal role in the transition from prostrate to erect growth during rice domestication (Jin et al., 2008; Tan et al., 2008; Zhang et al., 2023). *TAC1* is a major gene that controls the tiller angle in rice (Yu et al., 2007). Its homologous gene controls the leaf angle of maize (Ku et al., 2011). *LAZY1*, first identified in rice, causes changes in rice tiller angle by affecting the asymmetric distribution of auxin (Li et al., 2007; Yoshihara and Iino, 2007). *OsPIN2* controls gravitropism by finely regulating auxin transport, distribution and levels, and its overexpression leads to more tillers and a larger tiller angle compared with the wild type (Chen et al., 2012; Li et al., 2022a). In addition to auxin, other plant hormones such as ethylene, gibberellin and brassinosteroids, are also involved in branch (tiller) angle regulation (Edelmann, 2002; Nugroho et al., 2012; Vandenbussche et al., 2013; Wang et al., 2022b).

Currently, little is known about the mechanism of branch angle formation in rapeseed. Research methods for determining the branch angle of rapeseed are mainly based on quantitative trait loci (QTL) mapping and genome-wide association (GWAS) analysis. Using these methods, branch angle QTL have been localized on all chromosomes, except for chromosome C1, with chromosomes A3, A7 and C3 being the most distributed (Liu et al., 2016; Sun et al., 2016; Wang et al., 2016; Li et al., 2017; Shen et al., 2018; Zhao et al., 2022). Wang et al. detected a major QTL for branch angle on chromosome A6 using QTL-Seq, and identified the auxin synthesis-related gene *BnaYUCCA6* as a candidate gene (Wang et al., 2016). Zhao et al. identified a stably expressed QTL on chromosome C4 and screened the calcium-dependent protein CPK24, auxin response factor ARF10 and ABC-2 type transporter family protein ABCG3 as branch angle related proteins (Zhao et al., 2022). *PIN1*, *PIN2*, *LAZY1* and *ARF17* were identified as candidate genes by GWAS and RNA-Seq in rapeseed and may affect branch angle by participating in the asymmetric distribution of auxin (Wang et al., 2022b).

In this study, we obtained two parent materials, L7040 and L7043, with significant branch angle differences. A new branch angle QTL was mapped on chromosome C3 of *B. napus* by BSA and QTL-Seq. A total of 54 genes were found to have SNPs/InDels in this physical interval. RNA-seq was employed to further reveal transcriptome differences in branch angle formation in L7040 and L7043. Five candidate genes were involved in branch angle formation in *B. napus*. Our findings provide a new perspective for studying the molecular mechanisms and regulatory genes involved in branch angle formation in *B. napus*.

## Materials and methods

### Plant materials and rapeseed branch angle measurements

The parental materials, L7040 (♀) and L7043 (♂), are self-crossed F_11_ generation materials that possess great branch angle differences and are genetically stable. The L7040 material had a stable branch angle of 28-30 degrees, while L7043 was stable at 55-58 degrees. L7040 (P1) and L7043 (P2) were crossed to obtain the F_1_ generation, and then the F_1_ generation was used to generate an F_2_ segregating population by self-crossing. All materials were grown in experimental fields (26.50°N, 106.66°E) of the Guizhou Academy of Agricultural Sciences, Guiyang, China, with row spacing of 40 cm and plant spacing of 25 cm. Measurement of the rapeseed branch angle was based on the method described by Wang et al. (2022b). In brief, the first to fifth branch from top to bottom of each plant was taken at maturity. The branches along with some of the main stems were cut off and arranged in branch order on a level ground, photographed with a digital camera in vertical ground orthographic projection and then angular measurements were taken using the ruler tool of Photoshop CS6. The average of the angles of the first to fifth branches of each individual plant was taken as the branch angle of that individual plant.

### Bulked segregant analysis sequencing (BSA-seq)

The F_2_ segregating population and their parents (P1 and P2) were subjected to BSA-seq. Genomic DNA of rapeseed was extracted from the leaves using Plant Genomic DNA kit (TIANGEN, China) according to the manufacturer’s instructions. The DNA concentration was estimated using the Nanodrop 1000 spectrophotometer and subjected to 1.5% agarose gel electrophoresis to assess purity. Then, the DNA from the 20 plants with the smallest branch angle was equally mixed to form the S-pool, and that from the 20 plants with the largest branch angle formed the B-pool. The prepared S and B pool, and the DNA of the two parents were sent to Novogene (Beijing, China) for library construction. The constructed library was sequenced using an Illumina HiSeq PE150, and the sequencing depth was 20× for the progeny pool and 10× for the parents. After quality assessment, the sequencing data were compared to the reference genome of *B.napus* (http://www.genoscope.cns.fr/brassicanapus/data/) for SNP detection and annotation. Based on the SNP detection results, differences between the SNP indices of the two extreme progenies were calculated. Thresholds were determined based on sequencing depth, population type, and number of mixed-pool monocultures in each interval. The methods for calculating SNP-index of two pools and the derived Δ(SNP-index) were based on the description of Takagi et al. (2013).

### QTL analysis using InDel markers

A new population was constructed for QTL validation by randomly selecting 92 plants from the F_2_ segregating population containing 303 plants. After the QTL-seq analysis, indel markers were developed on chromosome C3 (not shown). Genetic maps were constructed using Joinmap 4.0 and QTL scanning was performed using WinQTLCart 2.5. The QTL analysis was performed using composite interval mapping (CIM) with a step size of 2 cM. Window size, scanning spacing, and spacing of neighboring QTLs were set to 10, 1, and 5 cM, respectively. Thresholds for the QTL were determined using a permutation test with 1000 repeated samples, and the level of significance was set at 0.05. QTL was considered to be present when the LOD value of ≥ 2.5.

### RNA sequencing and transcriptome analyse

The branch angle of *B.napus* was in the stable stage from 15 to 65 days after branching (Wang et al., 2022b). Therefore, we selected tissues from the adaxial (N) and abaxial (L) sides of the third branch nodes of L7040 (S) and L7043 (B) before they became stable (at the beginning of the flowering stage). Ten individual plants were mixed in a repeat, and each sample was taken in triplicate. The samples were frozen in liquid nitrogen and stored in an ultra-low temperature refrigerator until further use. Total RNA was extracted using the EZ-10 DNAaway RNA Mini-Preps Kit (Sangon, China) according to the manufacturer’s instructions. The total RNAs that passed the concentration and purity quality checks were sent to Biomarker Technology (Beijing, China) for cDNA library construction and transcriptome sequencing. A total of 12 libraries (SN-1, SN-2, SN-3, SL-1, SL-2, SL-3, BN-1, BN-2, BN-3, BL-1, BL-2, and BL-3) were constructed and sequenced using the Illumina NovaSeq 6000 platform based on the PE150 strategy. The clean reads were mapped to the ‘Zhongshuang11’ (‘ZS11’) reference genome (Song et al., 2020; Yang et al., 2023) using HISAT2 tools with default parameters. Gene expression levels were estimated as fragments per kilobase of transcript per million fragments mapped (FPKM). Differential expression analysis was performed using DESeq2, and the genes with |log2 (fold change)| ≥1 and adjusted FDR <0.01 were assigned as differentially expressed genes (DEGs). The GOseq R packages and KOBAS software were used for GO and KEGG enrichment analysis of the DEGs, respectively.

### Quantitative real-time PCR (qRT-PCR) analysis

The qPCR primers for the candidate genes were obtained from the BrassicaEDB database (https://brassica.biodb.org/) (Chao et al., 2020). Details of the primers used are listed in **Supplementary Table S1**. The EasyScript^®^ All-in-One First-Strand cDNA Synthesis SuperMix for qPCR (One-Step gDNA Removal) kit (TIANGEN, China) was used for reverse transcription. The qRT-PCR was performed using Bio-Rad CFX96 instrument as previously described (Jiang et al., 2020).

### Measurement of indole-3-acetic acid (IAA) content

The IAA content in the SN-1, SN-2, SN-3, SL-1, SL-2, SL-3, BN-1, BN-2, BN-3, BL-1, BL-2, and BL-3 tissues was determined using the ESI-HPLC-MS/MS method. Approximately 150 mg of fresh weight of sample was ground to powder in liquid nitrogen. The powder was accurately weighed and extracted with 1 mL of isopropanol-water-hydrochloric acid mixture containing 8 ng of IAA as internal standard. After shaking at 4°C for 30 min, 0.3 mL of dichloromethane was added to continue shaking for 30 min. Then centrifuged at 4°C, 13,000 rpm for 5 min, the lower layer organic phase was taken for nitrogen blow drying. The methanol containing 0.1% formic acid was utilized to re-solubilize and then centrifuged again at 4°C at 13,000 rpm for 10 min, and the supernatant was filtered utilizing a 0.22 µm filter membrane. The IAA was separated and detected using an Agilent Poroshell 120 SB-C18 analytical column (150×2.1 mm, 2.7 μm) and Agilent1290 high-performance liquid chromatography (HPLC) coupled with a triple quadrupole mass spectrometer (AB SCIEX-6500Qtrap MS/MS, Applied Biosystems, U.S.A.). IAA content was quantified on the basis of the ratio between the ion intensity and the internal standard. The IAA standard were purchased from Dr. Ehrenstorfer GmbH (Germany). The standard curve equation used for quantification in this study was Y=1.34281X+ 0.00648, R=0.99747.

## Results

### Genetic characterization of branch angle in *B.napus*

**Figure 1:**
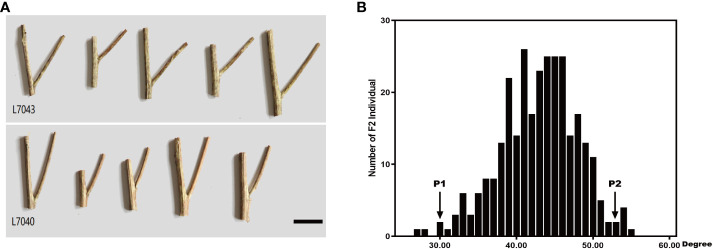
**(A)** Phenotypes of L7043 and L7040 with different branch angle, Bar=3 cm; **(B)** Frequency distribution of branch angle of 92 individuals in F2 population. P1:L7040; P2:L7043.

Rapeseed segregation populations L7040 and L7043 with small and large branch angles, respectively, were obtained through years of field self-crossing experiments. L7043 (P2), with an average branch angle of 52.8°, was much larger than L7040 (P1), which had an average branch angle of 30.13° (**Figure 1A**). The F_2_ population was obtained by crossing L7040 and L7043 and self-crossing. Statistical analysis showed that the branch angles of 297 individuals in the F_2_ population varied continuously and followed a normal distribution (AD=0.14, P=0.977) (**Figure 1B**). This result suggests that branch angle in *B. napus* is a quantitative trait.

### QTL for branch angle identified by QTL-Seq and InDel markers

**Figure 2:**
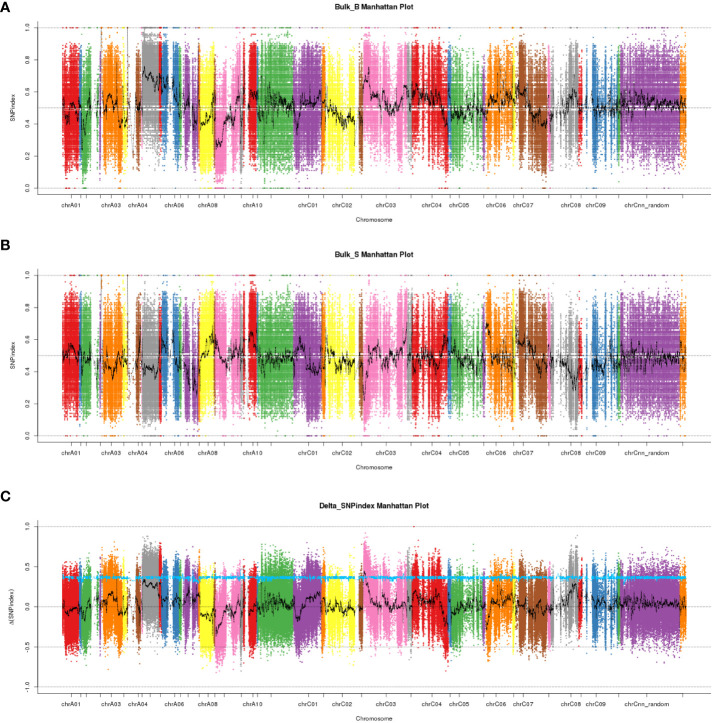
The manhattan plots of SNP-index and Δ(SNP-index) from QTL-seq analysis. **(A)** The manhattan plot of SNP-index in the B-pool. X-axis represents the position of 19 chromosomes and Y-axis represents the SNP-index. **(B)** The manhattan plot of SNP-index in the S-pool. X-axis represents the position of 19 chromosomes and Y-axis represents the SNP-index. **(C)** The manhattan plot of Δ(SNP-index). The Δ(SNP-index) graph was plotted with statistical confidence intervals under the null hypothesis of no QTL (P<0.05).

We performed high-throughput genomic DNA sequencing of the two parents (L7043 and L7040) and two pools (B-pool and S-pool). A total of 2,307,510 SNPs were detected in the two parents. Of these, 606,113 SNPs that were purely divergent between the two parents were used to calculate the SNP-index (frequency of SNPs) in the B-pool and S-pools (**Figures 2A, B**). The Δ(SNP-index) was shown in **Figure 2C**, and the 95% confidence level (blue line) was selected as the threshold for candidate interval screening based on 1,000 permutation tests. Candidate SNPs screening were first performed based on |Δ(SNP-index)| ≥ 0.7, and a total of 251 SNPs associated with branch angle were screened (**Supplementary Table S2**). These SNPs were mainly distributed on chrA03, chrA05, chrA09, chrC03, chrC04 and chrC08. Based on the annotation information of these SNPs, which causing non-synonymous mutations or stop gain or stop loss on exons were then selected as candidate SNPs, and the genes in which these SNPs are located were used as candidate genes. At last, a total of four SNPs resulting in non-synonymous variants were identified, which were derived from a total of four genes, three of which were on chrC03 and one on chrC08 (**Supplementary Table S2**). Based on this, the genomic location of the SNPs selected on chrC03 as the important candidate intervals.

**Figure 3:**
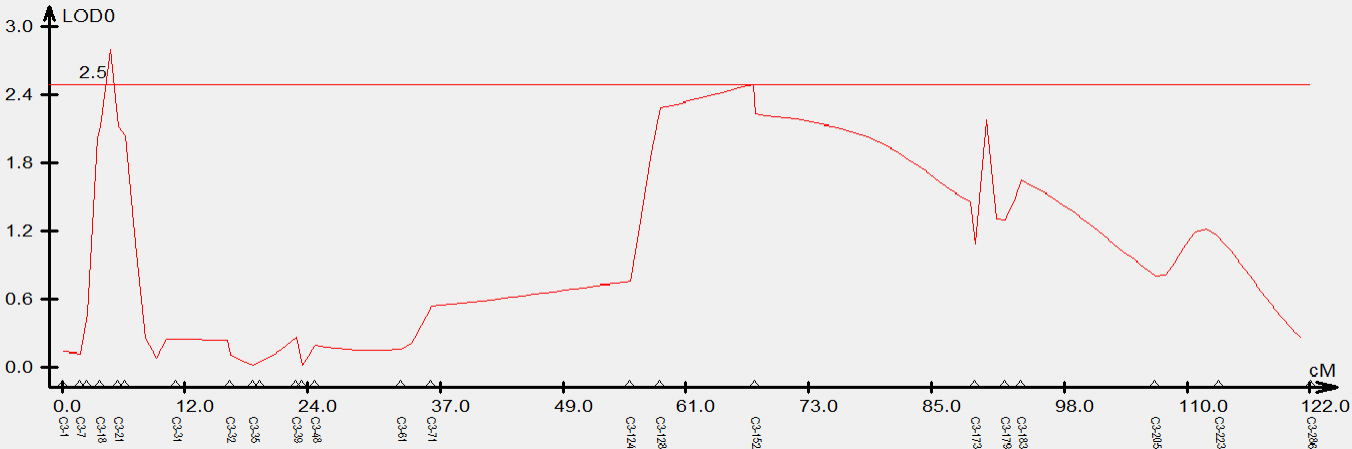
Genetic validation of branch angle QTL in *Brassica napus* chromosome C3 using an F2 population. The x-axis indicates the genetic distance between indel markers. The y-axis indicates the QTL LOD scores.

To validate the candidate intervals predicted by QTL-seq, traditional QTL mapping was performed on the F_2_ population of 92 individual plants. We detected 717,464 indel markers distributed across different chromosomal regions using high-throughput sequencing. Furthermore, 24 polymorphic markers located on chromosome C3 were used to genotype 92 F_2_ plants and the data were used for linkage analysis. A QTL was identified on chromosome C3 (C3: 1.54-2.65 Mb) using the ICIM, which had a LOD value larger than 2.5 and explained 14% of the phenotypic variation. This result was consistent with the △(SNP-index) obtained from QTL-Seq, while narrowing the localization interval. Based on this, we concluded that the QTL for the branch angle was located in the genomic interval of C3: 1.54-2.65 Mb (**Figure 3**).

### Comparative transcriptomic analysis

To further explore the molecular mechanisms of branch angle formation in rapeseed, a comparative transcriptomic analysis was performed. A total of 73.89 Gb of clean data were obtained from the 12 sequencing libraries, and the Q30 of each sample was greater than 92.75%. The mapping ratio of each sample to the reference genome ranged from 85.32% to 94.40% (**Supplementary Table S3**).

**Figure 4:**
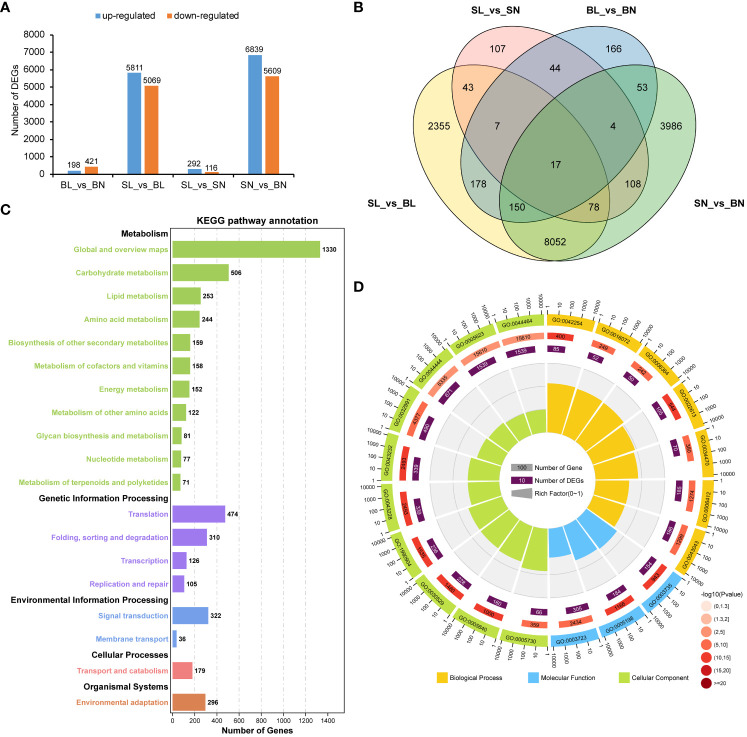
Transcriptomic analysis of branch angle in oilseed rape. **(A)** Statistical analysis of DEGs. BL vs. BN indicates a comparison between the branch’s abaxial and adaxial sides of the large-angle accessions. SL vs. BL refers to the comparison between small- and large-branch angle accessions on the abaxial side. SL vs. SN indicates a comparison between the abaxial and adaxial branches of the small-angle accessions. SN vs. BN refers to the comparison between small- and large-branch angle accessions on the adaxial side. **(B)** Venn diagram of the DEGs. **(C)** Top 20 enriched KEGG pathways of the DEGs overlapping in SL vs BL and SN vs BN. **(D)** Top 20 GO functional enrichment of the DEGs overlapping in SL vs BL and SN vs BN. The KEGG pathway and GO functional enrichment analysis were performed using the OmicShare tools (http://www.omicshare.com/tools).

DEG analysis showed that there were 619 differentially expressed genes between BL and BN, of which 198 were up-regulated and 421 were down-regulated. There were 408 DEGs between SL and SN, of which 292 were up-regulated and 116 were down-regulated. In contrast, there were 10,880 (5,811 up-regulated and 5,069 down-regulated) and 12,448 (6,839 up-regulated and 5,609 down-regulated) differentially expressed genes between SL and BL and SN and BN, respectively (**Figure 4A**). In addition, we also found that there were specific expressions of DEGs between each group, including 2,355 in SL_vs_BL, 107 in SL_vs_SN, 166 in BL_vs_BN, and 3,986 in SN_vs_BN (**Figure 4B**). We then focused on the co-expression DEGs between SL_vs_BL and SN_vs_BN. We performed KEGG and GO enrichment analysis for the 8,297 co-expressed DEGs. As shown in **Figure 4C**, they were enriched into a total of five KEGG categories. Most DEGs were related to metabolism, such as carbohydrate metabolism (506 DEGs), lipid metabolism (253 DEGs), amino acid metabolism (244 DEGs), biosynthesis of other secondary metabolites (159 DEGs), metabolism of cofactors and vitamins (158 DEGs) and energy metabolism (152 DEGs). In addition, there were also a large number of DEGs involved in genetic information processing, environmental information processing and cellular processes, including 474 DEGs in translation, 310 DEGs in folding, sorting and degradation, 322 DEGs in signal transduction and 179 DEGs in transport and catabolism. Furthermore, 296 DEGs were associated with environmental adaptation (**Figure 4C**, **Supplementary Table S4**). For the GO functional enrichment, the top 20 significantly enriched GO terms are shown in **Figure 4D**. Cell (GO:0005623) and cell part (GO:0044464) were the two GO terms with the highest number of enriched DEGs (1,539). For the molecular function in the GO categories, RNA binding (GO:0003723), structural molecule activity (GO:0005198), and structural constituent of ribosome (GO:0003735) were the three most significantly enriched GO terms, with 305, 184, and 154 DEGs, respectively. For the biological process in the GO categories, among the top 7 GO terms with the most significant enrichment, peptide biosynthetic process (GO:0043043), translation (GO:0006412) and ribonucleoprotein complex biogenesis (GO:0022613) contained the largest number of DEGs, with 186, 185 and 105 respectively (**Figure 4D**; **Supplementary Table S5**).

### Identification of candidate genes by QTL and RNA-seq

Based on the QTL results, we identified 54 genes with functional annotations in the physical interval (C3: 1.54-2.65 Mb). A total of 51/54 genes had insertion/deletion mutations, and 4/54 genes had SNP differences between two parents. Notably, *BnaC03G0051800ZS* exhibited both InDel and SNP differences. **Supplementary Table S6** shows the variant elements for each gene, variant start and termination positions, base type and genotype in the assay samples, and detailed gene annotations.

**Figure 5:**
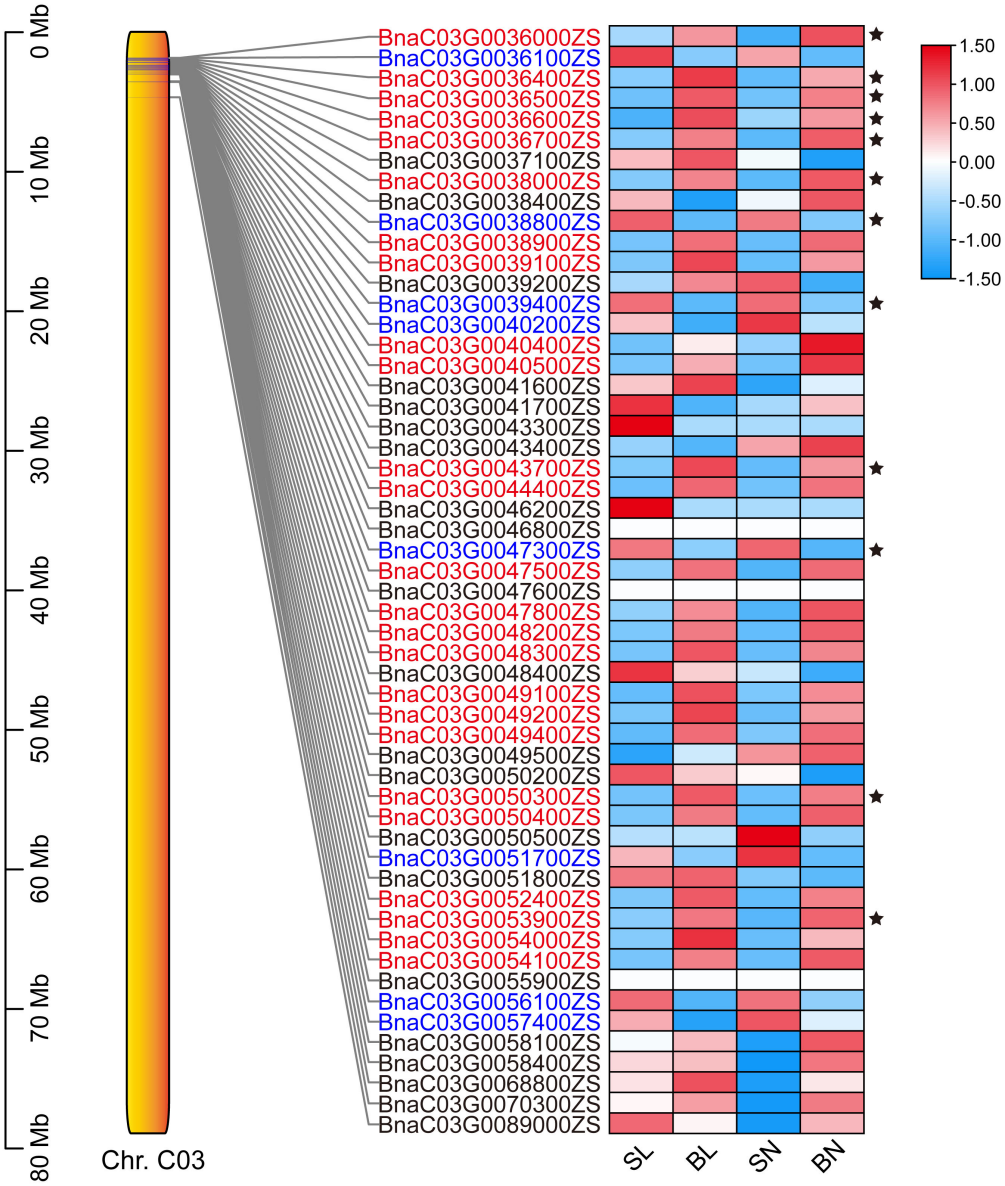
The expression patterns of the 54 candidate genes based on RNA-seq. The position distribution map of genes on chromosomes and the heat map of expression were drawn using TBtools (Chen et al., 2023). The 12 DEGs were marked with black five-pointed stars.

The expression patterns of the 54 genes were further analyzed based on transcriptome data. As shown in **Figure 5**, a total of 33 genes had regular expression patterns in the branching angle difference samples (S and B). The expression levels of 25 of them (red font in **Figure 5**) were lower in S samples than in B samples of the same tissue, while the remaining eight (blue font in **Figure 5**) were the opposite. Among them, 12 genes with significant level of difference were selected as new candidate genes (**Figure 5**, **Table 1**). They were further functionally annotated with *Arabidopsis* homologous genes, and the results showed that *BnaC03G0038000ZS* and *BnaC03G0043700ZS* may encode ribosomal proteins (**Table 1**). The homolog of *BnaC03G0036600ZS* in *Arabidopsis* is *AT5G07920*, which encodes diacylglycerol kinase 1, a key enzyme involved in hormone regulation and salt stress signal transduction (Derevyanchuk et al., 2019). *BnaC03G0036400ZS* homolog gene in *Arabidopsis* is *AT5G61210* (*AtSNAP33*), which performs disease-resistance functions via the SA pathway after pathogen inoculation (Wick et al., 2003). *BnaC03G0036700ZS* homologue of *Arabidopsis* gene *AT5G08020* encodes a homolog of replication protein A, which is involved in DNA damage repair (Aklilu et al., 2020). *BnaC03G0047300ZS* homolog gene *AT5G10430* encodes arabinogalactan protein 4 (JAGGER), which is involved in reproductive processes such as signaling pathways, leading to blocked pollen tube attraction in *Arabidopsis* (Pereira et al., 2016). *BnaC03G0050300ZS* homolog *AT5G11110* (*AtSPSA2*) in *Arabidopsis* is involved in carbon partitioning and the drought response (Bagnato et al., 2023).

**Table 1:** Candidate genes screened by QTL and RNA-Seq.

Candidate gene		Homologous in *Arabidopsis*	log2FC	
	Accession number	Functional description	SL vs BL	SN vs BN
BnaC03G0036000ZS	AT5G07810	SNF2 domain-containing protein/helicase domain-containing protein/HNH endonuclease domain-containing protein;	1.12	2.08
BnaC03G0036400ZS	AT5G61210	SNP33; soluble N-ethylmaleimide-sensitive factor adaptor protein 33	2.62	2.33
BnaC03G0036500ZS	AT5G07910	Leucine-rich repeat (LRR) family protein	1.60	1.28
BnaC03G0036600ZS	AT5G07920	Diacylglycerol kinase 1	2.00	1.02
BnaC03G0036700ZS	AT5G08020	Replication protein A 70 kDa DNA-binding subunit B	1.17	1.43
BnaC03G0038000ZS	AT5G08180	Ribosomal protein L7Ae/L30e/S12e/Gadd45 family protein	1.10	1.36
BnaC03G0038800ZS	AT5G08290	Thioredoxin-like protein YLS8	-1.77	-1.52
BnaC03G0039400ZS	AT5G08350	GEM-like protein 4/ABA-responsive protein-like protein	-2.73	-2.34
BnaC03G0043700ZS	AT5G09770	Ribosomal protein L17 family protein	3.43	3.55
BnaC03G0047300ZS	AT5G10430	Encodes arabinogalactan-protein (AGP4).	-1.91	-2.64
BnaC03G0050300ZS	AT5G11110	Sucrose phosphate synthase 2	1.65	1.48
BnaC03G0053900ZS	AT5G11880	meso-diaminopimelate decarboxylase 1	1.16	1.39

### Candidate genes validation

**Figure 6:**
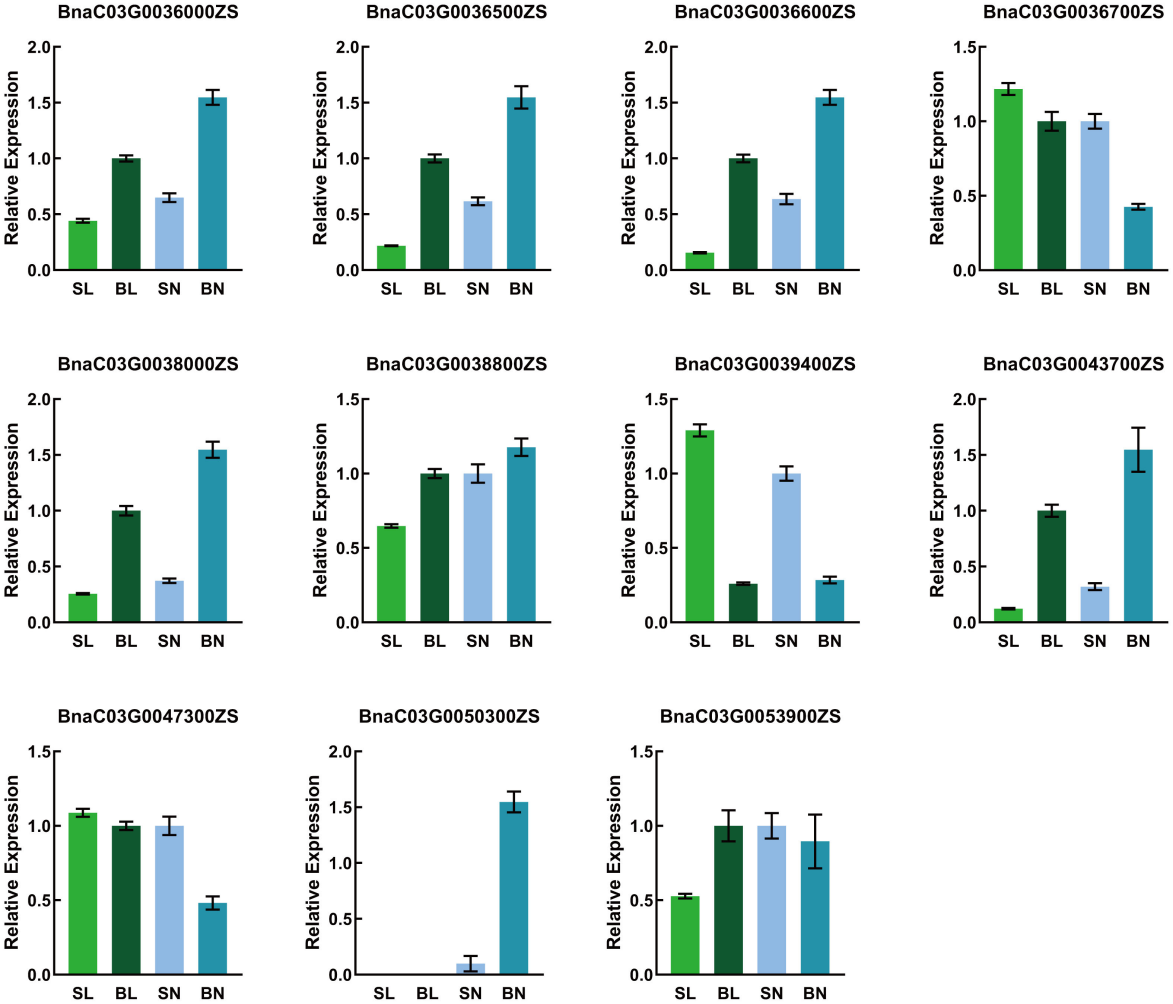
Relative expression of candidate genes in the SN, SL, BN, and BL as detected by qRT-PCR.

To verify the expression of the candidate genes in the SN, SL, BN and BL samples, we performed qRT-PCR on these 12 genes. As shown in **Figure 6**, 11 genes were detected, whereas *BnaC03G0036400ZS* was not detected because its expression was too low. The expression trends of the candidate genes in qRT-PCR were consistent with those in RNA-Seq, except for *BnaC03G0036700ZS* and *BnaC03G0038800ZS*. The expression levels of *BnaC03G0036000ZS*, *BnaC03G0036500ZS*, *BnaC03G0036600ZS*, *BnaC03G0038000ZS* and *BnaC03G0043700ZS* in samples with small branching angles were significantly lower than those in samples with large branching angles, while the opposite was true for *BnaC03G0039400ZS*. This consistent differential expression pattern indicated that they may be involved in the regulation of branch angle formation. Noteably, *BnaC03G0050300ZS* was only detected in the adaxial samples, indicating that the gene had a tissue-specific expression pattern that predicted the differentiation of its function on the adaxial and abaxial sides of the branches.

### Analysis of IAA content and expression pattern of key genes of IAA synthesis and signal transduction pathway

**Figure 7:**
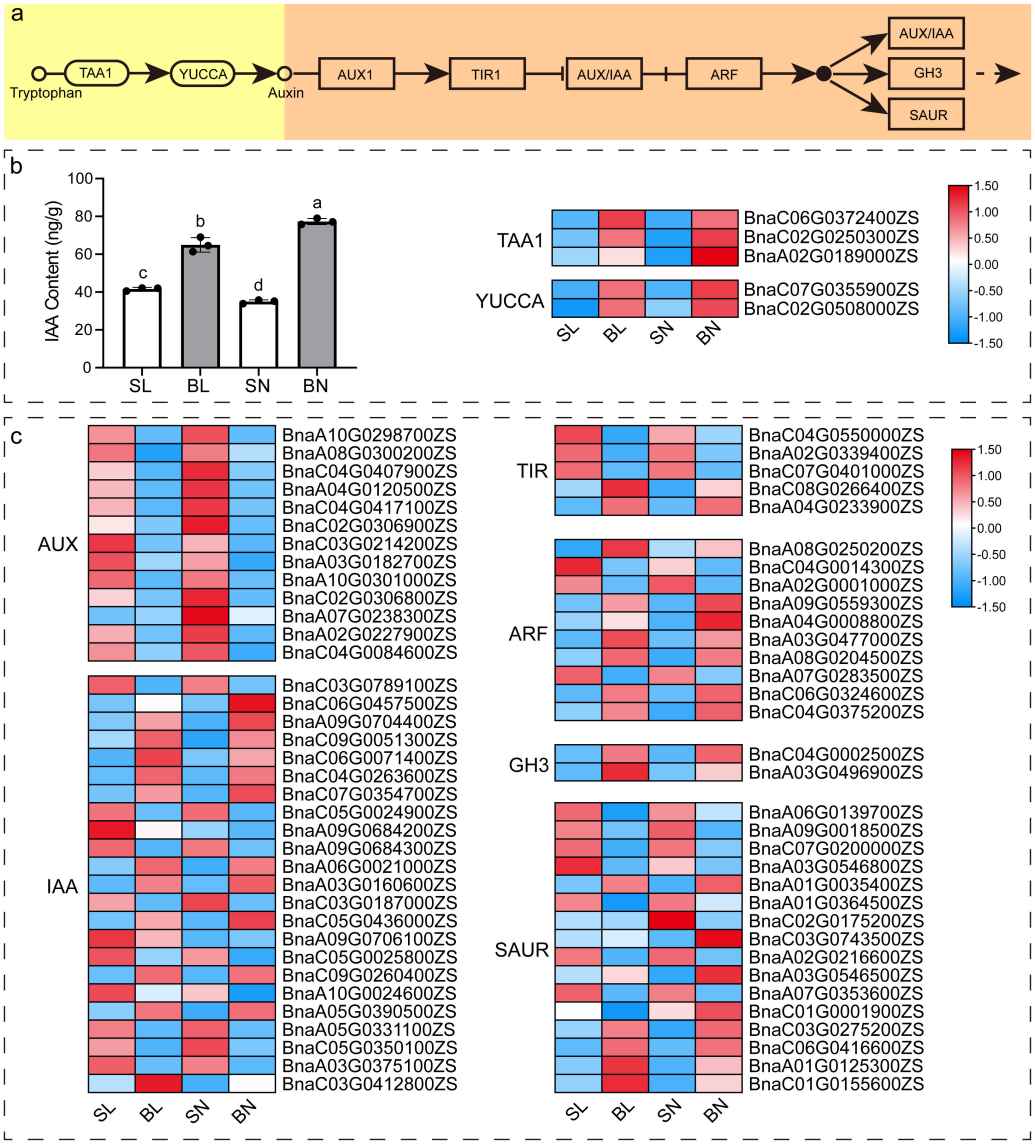
IAA content and expression pattern of key genes of IAA synthesis and signal transduction pathway. **(A)** Diagram of the major pathways of IAA synthesis (Won et al., 2011) and signaling pathways (https://www.kegg.jp/entry/map04075) in plants. **(B)** IAA content and heat map of expression of key genes of IAA synthesis pathway in four tissues. The different letters on the bar graph indicate significant differences at p < 0.05 according to Duncan’s test. TAA1: tryptophan aminotransferase of *Arabidopsis* 1; YUCCA: YUC flavin monooxygenase. **(C)** Heat map of the expression of key genes of the IAA signaling pathway. AUX: auxin influx carrier; IAA, auxin-responsive protein; TIR, transport inhibitor response; ARF, auxin response factor; GH3, auxin responsive GH3 gene family; SAUR, SAUR family protein.

As previous studies (Wang et al., 2016; Cheng et al., 2017; Shen et al., 2018; Li et al., 2019; Wang et al., 2022b) have shown that auxin signaling pathway plays an important role in the regulation of branching angle in rapeseed, we detected the content of IAA in the four tissues with transcriptomic sequencing, and further analyzed the expression patterns of key genes of auxin synthesis and signaling pathway. As shown in **Figure 7**, there was a significant difference in the content of IAA in these four tissues, with the content in both tissues at the small branching angle being lower than that at the large branching angle. In terms of the adaxial and abaxial tissues, the content of abaxial tissue in small branching angles was higher than that in adaxial tissue, while the content of large branching angles was just the opposite (**Figure 7B**). We identified 5 DEGs in the IAA synthesis pathway from our transcriptome data, including three *TAA1* members and two *YUCCA* members, and their expression patterns were approximately the same as the IAA content. In addition, a total of 69 DEGs were obtained in auxin signaling pathway (**Figure 7C**). Interestingly, these DEGs showed differences between samples of the same tissue parts in different branching angle materials. For example, the expression of all 13 AUX members at the same tissue parts was higher in the small branching angle material than in the large branching angle, whereas the opposite was observed for the two members of GH3. However, not all members of the same gene family showed consistent expression patterns. For example, for the 23 members of IAA, 11 of them showed expression patterns consistent with AUX, while the remaining 12 showed expression patterns consistent with GH3. This phenomenon was also presented in TIR, ARF, and SAUR families.

## Discussion

The branch angle of rapeseed has been identified as a quantitative trait in this study and previous reports. At present, quantitative traits are mainly studied by QTL mapping and GWAS. Traditional QTL mapping methods require the construction of a large number of mapping populations and molecular markers, which is a heavy workload and time consuming, severely limiting the development of QTL mapping (Merrick et al., 2022). With the development of high-throughput sequencing technology, the mixed pool of extreme parents and offspring has been sequenced by combining it with BSA. The differential SNPs and indels obtained were analyzed, and a genetic map was constructed to quickly perform QTL mapping (Li and Xu, 2022).

Currently, most plant populations used for branch angle mapping are natural or DH lines. Although QTL can be successfully identified using this method, the number of QTL is high and scattered. For example, Shen et al. genotyped a DH population containing 208 lines using the 60 K Brassica Infinium SNP array, constructed a genetic map, and a total of 17 QTLs were mapped (Shen et al., 2018). Liu et al. performed a GWAS analysis of branch angles using a natural population containing 143 lines and mapped 25 significant QTLs on chromosomes A2, A3, A7, C3, C5 and C7 (Liu et al., 2016). Although these studies help understand the molecular mechanism of branch angle formation, there are too many mapping intervals, thus impeding the screening of candidate genes. However, Wang et al. used Purler and Huyou 19, two parents with significant differences in branch angle, to fine map the F_2_ population using BSA combined with QTL-seq technology, and successfully screened the branch angle-related gene *BnaYUCCA6* (Wang et al., 2016). Based on this, we believe that QTL mapping using self-crosses with significant differences in branch angles achieves better results than using natural populations. The parents, L7043 and L7040 used in this study were self-crossed F_11_ generations with large differences in branch angles and stable genetic characteristics. In this study, a QTL locus was successfully identified on chromosome C3 using BSA-seq and QTL mapping. Some QTLs/QTNs for branch angle in *B. napus* localized to chromosome C3 have been reported (Liu et al., 2016; Sun et al., 2016; Li et al., 2017; Shen et al., 2018; Wang et al., 2019; Hu et al., 2022; Zhao et al., 2022; Wang et al., 2022b), but our intervals were not consistent with these finding. It suggests that, C3 chromosome is the key to the formation of rapeseed branching angle, but, it also indicates that we have mapped a new QTL controlling rapeseed branching angle.

Previous studies have shown that branch angle formation is related to the gravitational response of plants (Yoshihara and Spalding, 2017; Xia et al., 2021; Kawamoto and Morita, 2022; Li et al., 2022b). In a study on tiller angle in rice, it was found that after sensing gravity, asymmetric distribution of auxin occurred in rice through gene network signal transduction. The lengths of the cells on both sides of the stem changed, leading to a change in the tiller angle. The difference in the growth rate between the adaxial and abaxial sides leads to a difference in the branch angle (Wang et al., 2022a). Therefore, in this study, L7043 and L7040 were sampled for RNA-seq analysis on the adaxial and abaxial sides, respectively. The DEGs between the same tissues of two materials were much more than that between the different tissues of the same material, indicating that the difference of rapeseed branching angle was more affected by genetic background. The KEGG results showed that the DEGs responsible for the difference in branch angle between L7040 and L7043 were mainly enriched in the ribosome, plant-pathogen interaction, plant hormone signal transduction, and starch and sucrose metabolism pathways. These results were consistent with those of previous studies and implies that plant hormones play an important role in branch angle formation (Cheng et al., 2017; Wang et al., 2022b). However, we also found some inconsistent results with the previous studies. For example, Wang et al. found that the content of IAA in rapeseed with small branch angle was lower than that in rapeseed with large branch angle of the same tissue (Wang et al., 2016), while our results were contrary to this. In contrast, the expression patterns of IAA synthesis pathway genes in both studies were consistent with the content of IAA, foreshadowing that the difference in IAA content between the two studies may be related to the different sampling periods and tissues.

Based on the QTL and RNA-seq results, 12 candidate genes may play an important role in branch angle formation. These are structural proteins or catalytic enzymes that function through signal transduction pathways. The known candidate genes identified on chromosome C3 of *B.napus* that is involved in branch angle formation is *LAZY1* (*BnaC03G0065700ZS*), SAUR-like auxin-responsive protein (*BnaC03G0149900ZS*, *BnaC03G0166500ZS*), *IAA19* (*BnaC03G0412800ZS*), *ADS3/FAD5*(*BnaC03G0414300ZS*, *BnaC03G0414500ZS*), *WSIP2* (*BnaC03G0414600ZS*) (Liu et al., 2016; Shen et al., 2018). *LAZY1* is a well-known gene that controls tiller angle in rice through asymmetric auxin distribution caused by polar auxin transport (Li et al., 2007). *SAUR* and *IAA19* are involved in auxin synthesis, response, and influx (Tatematsu et al., 2004; Kinoshita et al., 2023). *ADS3/FAD5* encodes plastidic palmitoyl-monogalactosyldiacylglycerol D7 desaturase, which is responsible for the synthesis of 16:1 fatty acid from galactolipids and sulfolipids (Smith et al., 2013). *WSIP2* is involved in physiological damage and plant immunity (Griebel et al., 2023). It is thus clear that the regulation of the formation of branching angles in rapeseed is complicated. Although these known genes were involved in different pathways, most of them showed consistent differential expression in large branching angle tissues and small branching angle tissues (Wang et al., 2022b). Based on this phenomenon, we further screened six members with the same expression pattern as described above as candidate genes. It is noteworthy that although the candidate genes we identified are of unknown function and belong to the new candidate genes involved in branch formation in rapeseed, they exhibit sequence variants between the two parents. It indicates that molecular markers can be subsequently developed for functional genes identification based on their sequence variants. In addition, most of these variants occur in the promoter region, predicting that their differential expression patterns may be related to the different regulatory effects received. However, we did not perform a functional verification of these candidate genes in this study. We intend to further explore their influence on branch angle formation through overexpression, inhibition and knockout.

In conclusion, in this study, we constructed an F_2_ segregating population using two trait-stable genetic parents with extreme differences in branching angle, and mapped a major QTL on chromosome C3 (C3:1.54-2.65 Mb) associated with branching angle in rapeseed by BSA-seq and QTL mapping. A comparison with previous studies suggested that this is a new QTL. Six candidate genes were identified by RNA-seq and validated by qPCR. Our results provide new insights into the mechanisms of branching angle formation in rapeseed.

## Data availability statement

The datasets presented in this study can be found in online repositories. The names of the repository/repositories and accession number(s) can be found in the article/**Supplementary Material**.

## Author contributions

HJ: Conceptualization, Data curation, Funding acquisition, Writing – original draft, Writing – review & editing. SL: Conceptualization, Formal analysis, Funding acquisition, Methodology, Writing – original draft. LC: Formal analysis, Investigation, Methodology, Software, Writing – original draft. FL: Formal analysis, Investigation, Writing – original draft. YZ: Investigation, Validation, Writing – original draft. CZ: Formal analysis, Investigation, Writing – review & editing. HX: Investigation, Project administration, Writing – review & editing. RT: Investigation, Writing – review & editing. BY: Investigation, Writing – review & editing. LW: Investigation, Writing – review & editing.
